# Revealing genetic variation of *Actinobacillus pleuropneumoniae* Korean isolates using whole genome sequence analysis

**DOI:** 10.71150/jm.2512010

**Published:** 2026-04-21

**Authors:** Eun-Seo Lee, Su Min Kyung, Jun Ho Lee, Xi-Rui Xiang, Han Sang Yoo

**Affiliations:** 1 Department of Infectious Diseases, College of Veterinary Medicine, Seoul National University, Seoul 08826, Republic of Korea; 2 BK21 FOUR Future Veterinary Medicine Leading Education and Research Center, College of Veterinary Medicine, Seoul National University, Seoul 08826, Republic of Korea; 3 Reseach of Institute for Veterinary Science, College of Veterinary Medicine, Seoul National University, Seoul 08826, Republic of Korea

**Keywords:** *Actinobacillus pleuropneumoniae*, whole-genome sequencing (WGS), serotypes, Apx toxins, AMR genes

## Abstract

*Actinobacillus pleuropneumoniae* (APP) is the etiological agent of porcine pleuropneumoniae (PP), a high contagious respiratory disease with significant impact on the swine industry in both clinically and economically. Despite of the several attempts to control APP, the emergence of novel serotypes and antimicrobial resistance (AMR) strains highlights the importance of monitoring the genetic characteristics of APP at single nucleotide level. Despite the importance of genomic surveillance of APP to develop effective control strategies, genetic information on the recent Korean isolates of APP is not available at whole genome level. Therefore, in this study, six APP strains were isolated from porcine lungs with characteristic lesions of PP from 2022 to 2024. And their whole genomic sequences, serotypes, virulence factors, and AMR traits were investigated using combined short- and long-read sequencing methods. In silico PCR serotyping identified the isolates as serotype 1, 7, and 15, while one isolate was non-typeable. Multiple AMR genes including *Hinf_PBP3_BLA*, *Ecol_EFTu_PLV*, *tet(B)*, *tet(O)*, *tetR*, *sul2*, *aph(3'')-Ib*, *aph(6)-Id*, and *aph(3')-Ia* were detected. Also, these genes were located with adjacent to mobile genetic elements, suggesting the possibility of horizontal gene transfer. Phylogenetic comparison with 40 global APP complete genomes, presented that Korean isolates were closely related with China and Switzerland strains. This study provides the whole genome sequences based genetic characterization on the recent Korean isolates of APP, and this study emphasizes that continuous monitoring of APP genomic variation to support effective control of porcine pleuropneumoniae.

## Introduction

APP is the Gram-negative, encapsulated rod shape bacterium which causes PP, a severe respiratory disease in pigs (Sassu et al., 2018; Vilaró et al., 2024). APP has been classified into 19 serovars based on capsular polysaccharides and lipopolysaccharide O-antigen and 2 biovars based on the NAD dependency (To et al., 2024). Geographical and temporal difference have been shown in distribution of APP serotypes. Although APP infection induces a variety of clinical forms, most of the infections progress as acute form, which is characterized by hemorrhagic necrotizing pneumoniae and fibrinous pleuritis with high morbidity and mortality (Chiers et al., 2002). Therefore, APP has been considered as one of the most important diseases in swine industry (Gale and Velazquez, 2020).

APP produces exotoxins, known as RTX (repeats-in-toxin) toxins; ApxI, ApxII, ApxIII, and ApxIV. Each toxin has difference in hemolytic and cytolytic activities (Sassu et al., 2018). ApxI is strongly hemolytic and cytotoxic, ApxII is relatively hemolytic and cytotoxic. ApxIII is strongly cytotoxic but non-hemolytic. However, ApxIV is highly specific to APP and expressed only after infection in pigs (Beck et al., 1994).

Although several predisposing factors are involved in APP outbreaks, vaccines and antimicrobial agents have been widely used as the most common control measures for PP. However, the effectiveness of these control measures has recently been challenged by the serotype-specific immunity of APP and the emergence of antimicrobial resistance.

Although there is not common in nationwide control program for PP, the economic and clinical importance of this disease makes it essential to establish nationwide control measures through continuous monitoring (Guitart-Matas et al., 2022). Above all, in establishing control measures, it is very important to know the characteristics of APP, such as serotypes, genotypes, occurring in the region.

The current methods used to analyze APP serotypes, genetic characteristics are now facing new challenges (Angen et al., 2025). Therefore, applying cutting-edge method is essential to address these emerging issues. Genetic variation in serotypes related molecules, virulence factors and other phenotypic characteristics should be assessed at the SNP level through whole-genome sequencing (WGS) (Quainoo et al., 2017). Furthermore, WGS analysis might be used to predict the severity of PP by analyzing genes encoding virulence factors including Apx toxins. Also, the WGS analysis could be applied to epidemiological approach of AMR through the verification of AMR patterns and their acquisition pathways by analyzing the gene transfer elements such as plasmids, insert element genes, etc. (Vilaró et al., 2024; Zhu et al., 2024).

However, to date, public announcement of whole genome sequence of APP Korean isolate is not available. Therefore, the whole genome sequencing of six APP isolates from pig showing typical clinical signs of PP was carried out. With that information, the features of APP Korean isolates were characterized to clarify the epidemiological traits through comparative analysis with strains from around the world.

## Materials and Methods

### Isolation and identification of bacterial strains

Lung swabs were collected from 2022 to 2024 from pigs with typical PP lesions raised on commercial farms in Korea. Upon arrival at the laboratory, the samples were immediately inoculated onto brains heart infusion (BHI) agar (Difco, Becton Dickinson, USA) supplemented with 15 μg/ml nicotinamide-adenine dinucleotide (NAD; Sigma) and chocolate agar (Synergy Innovation Co., Korea). After overnight incubation of the plates at 37°C with 5% CO_2_, suspect colonies of APP were picked up and sub-cultured. The suspected isolates were identified using a matrix-assisted laser desorption ionization-time of flight-mass spectrometry (MALDI-TOF-MS) biotyper (Bruker Daltonik GmbH, Germany). After the identification, APP isolates were cultured on a Chocolate agar and amplified in Brain heart infusion (BHI) broth (Difco, Becton Dickinson, USA) with supplemented with 15 μg/ml NAD at 37°C with 5% CO_2_.

### Phenotypic characterization and growth patterns

To characterize the phenotypic properties and growth patterns of the APP isolates, cultivation on various media and growth curve assays were performed using reference serotype 2 and 5 strains. For the phenotypic characterization, the isolates were inoculated onto various media including blood agar, chocolate agar, BHI agar, MacConkey agar (Difco, Becton Dickinson, USA), and EMB agar (Difco, Becton Dickinson, USA). Growth morphology was assessed after 24 h of incubation at 37°C in 5% CO_2_ atmosphere. Additionally, for the growth curve analysis, all strains were cultured in NAD-supplemented BHI broth. The optical density at 600 nm (OD_600_) was measured at predetermined intervals using the VersaMax absorbance microplate reader (Molecular Devices, USA) to monitor replication efficiency. All growth assays were conducted in triplicate, and results were expressed as the Mean ± SD.

### Whole genome sequencing of APP Korean isolates

For whole genome sequencing, bacteria isolates were retrieved from overnight broth cultures and full genomic DNA was prepared with MagAttract HMW DNA kit (Qiagen). For high quality genome sequencing and de novo assembly, short and long read sequencing were performed. Short-read sequencing was performed using an Illumina NovaSeq 6000 (Illumina, USA) platform following a paired-end 2 × 150-bp protocol. The Oxford Nanopore (Oxford Nanopore Technologies, UK) platform was employed for long-read sequencing. De novo assembly was conducted using Flye v2.9. The quality of the sequencing, mapped reads and the result of Benchmarking Universal Single-Copy Orthologs (BUSCO) were analyzed (Tables S1 and S2). Whole genome sequence of APP Korean isolates was deposited into GenBank.

### Analysis of genetic characteristics of APP Korean isolates

Presence of toxin genes identified by in silico analysis were confirmed by PCR amplification of those specific genes. Toxin typing was done by multiplex polymerase chain reaction (mPCR) (Rayamajhi et al., 2005). Genes related with virulence factors and mobile genetic elements were annotated by using Prokka server v1. 14.6 (Kyung et al., 2023) and NCBI Prokaryotic Genome Annotation Pipeline (PFAP) (Tatusova et al., 2016). Antibiotics resistant genes were identified using Comprehensive Antibiotic Resistance Database (CARD) server and ResFinder 4.7.2 version (https://genepi.food.dtu.dk/resfinder) (Ali et al., 2021). Based on these information, the high-quality zoomable genetic circular maps are visualized by using CGView tool (Stothard and Wishart, 2005). The complete sequence were serotyped in silico using UniPro UGENE in silico PCR tool (Okonechnikov et al., 2012) and previously reported primers were used (Bossé et al., 2014; Ito and Sueyoshi, 2015).

### Phylogenetic analysis of APP Korean and global isolates

For comparative whole genome analysis, 40 APP whole genome datasets were gathered from NCBI GenBank database. The genome of APP stain 4074 available at NCBI (GenBank accession number CP029003) was used as a reference for the analysis. Maximum likelihood (ML) phylogenetic tree was constructed with 1000 bootstrap values using MEGA-11 software and displayed on the interactive tree of life (iTOLs). Also, the gene distributions of APP characteristics including Apx toxins, virulence factors and AMR genes were identified with the same server above. And this information was visualized on the heat map using the iTOLs program.

### Minimum inhibitory concentrations of APP Korean isolates

To determine the relationship between the presence of antibiotic resistance genes and actual antibiotic resistance expression, the minimum inhibitory concentrations (MICs) of seven antibiotics- tetracycline, doxycycline, minocycline, sulfamethoxazole, streptomycin, kanamycin, and neomycin- were determined for each isolate. The MIC tests were performed using the broth microdilution method according to the Clinical and Laboratory Standards Institute (CLSI) VET01 guidelines (CLSI, 2018). The MIC results for APP isolates were interpreted according to the clinical breakpoints defined in CLSI (CLSI, 2018). For instance, the resistance breakpoints for tetracycline were set at ≥ 2 μg/ml. For antibiotics which APP specific breakpoints were not provided in the CLSI guidelines, only the MIC values were reported to analyze their correlation with resistance genes.

## Results

### General genomic features of selected isolates

**Fig. 1.**
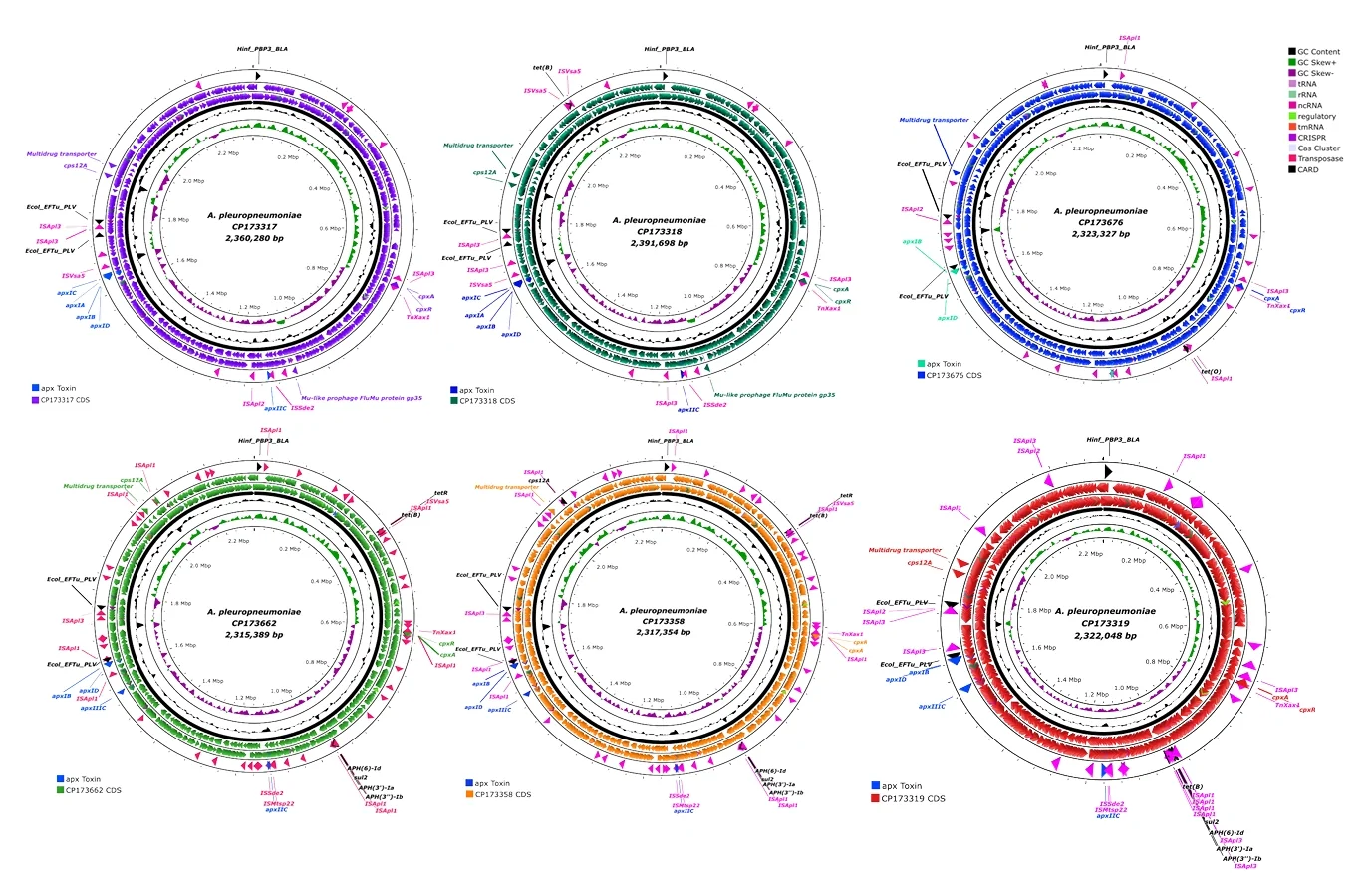
Circular map of APP isolates. All circular map of APP isolated in this study, were generated by using CGView server to visualize their structural and functional features. Each map illustrates the genomic features of APP isolates. From the innermost to the outermost ring: (1) GC skew, (2) GC content, and (3) coding sequences (CDS) colored according to the specific strain. At the outer edge of each map, key functional genes are annotated and color-coded for clarity. The blue labels indicate Apx toxin genes, while pink labels highlight mobile genetic elements such as transposases. Lastly, the antimicrobial resistant (AMR) genes are presented in black labels. Abbreviations: APP, *Actinobacillus pleuropneumoniae*; AMR, antimicrobial resistance; CDS, coding sequence; GC, guanine-cytosine; IS, insertion sequence.

Six strains of APP were isolated from lung of pigs showing typical clinical signs of PP. Whole genome sequencing was done with those strains. General genomic features and location of important genes in the chromosome of the six APP isolates are shown in Fig. 1 and Table 1, respectively. Most of the isolates were similar in genome size, GC content, and CDS encoding sites even though there was a little bit difference depending on the year of isolation (Table 1). However, the number of antibiotics resistance genes has been steadily increased. Whole genome sequences of recent Korean isolates of APP are available in NCBI GenBank data base (Table 1). By in silico analysis of *cps* gene clusters of those isolates, two isolates (APSW223 and APSW224) in 2022, one isolate (APSW232) in 2023 and other two isolates (APSW234 and APSW235) were identified into serotype 1, 7, and 15, respectively. However, APSW241 isolated in 2024 were not able to be serotyped.

### Phenotypic growth characteristics and morphology

The phenotypic growth rates of the isolates were consistent with those of the reference strains. All tested strains showed robust growth on chocolate agar and NAD-supplemented BHI agar forming small, circular, smooth, and grayish colonies after 24 h. While no growth was observed on blood agar and MacConkey agar. Furthermore, the colony size on EMB agar was limited compared to the size on chocolate and BHI agar. Thus, it was insufficient for a definitive morphological assessment (Table S5). In the growth curve analysis, all six isolates displayed growth patterns similar to those of the reference serotypes 2 and 5. The strains entered the exponential log phase within 2 h of incubation and reached the stationary phase after 6 h (Fig. S1).

### Genetic characteristics of the APP Korean isolates

Initially, the analysis of toxin-related factors revealed that all isolated APP strains harbored *apxIB*, *apxID*, and *apxIIC* genes and APSW223 and APSW224 strains harbored additional *apxIA* and *apxIC* genes (Table 2). APSW234, APSW235, and APSW241 strains harbored *apxIIIC* gene (Table 2). The transposase genes were found to be adjacent to the toxin genes through analysis of circular maps (Fig. 1). Specifically, the mu-like prophages FluMu protein *gp35* gene was identified in the APSW223 and APSW224 isolates (Table 2). Regarding the gene cluster encoding biosynthesis of capsular polysaccharide, *cps 12A* gene was identified in all isolates except APSW232 strain, and all isolates were carrying *cpx A* and *cpx R* genes. *Hinf_PBP3_BLA* and *Ecol_EFTu_PLV* genes were detected in all isolates, and more than three resistance genes were identified in all strains except APSW223 (Tables 2 and 3).

**Fig. 2.**
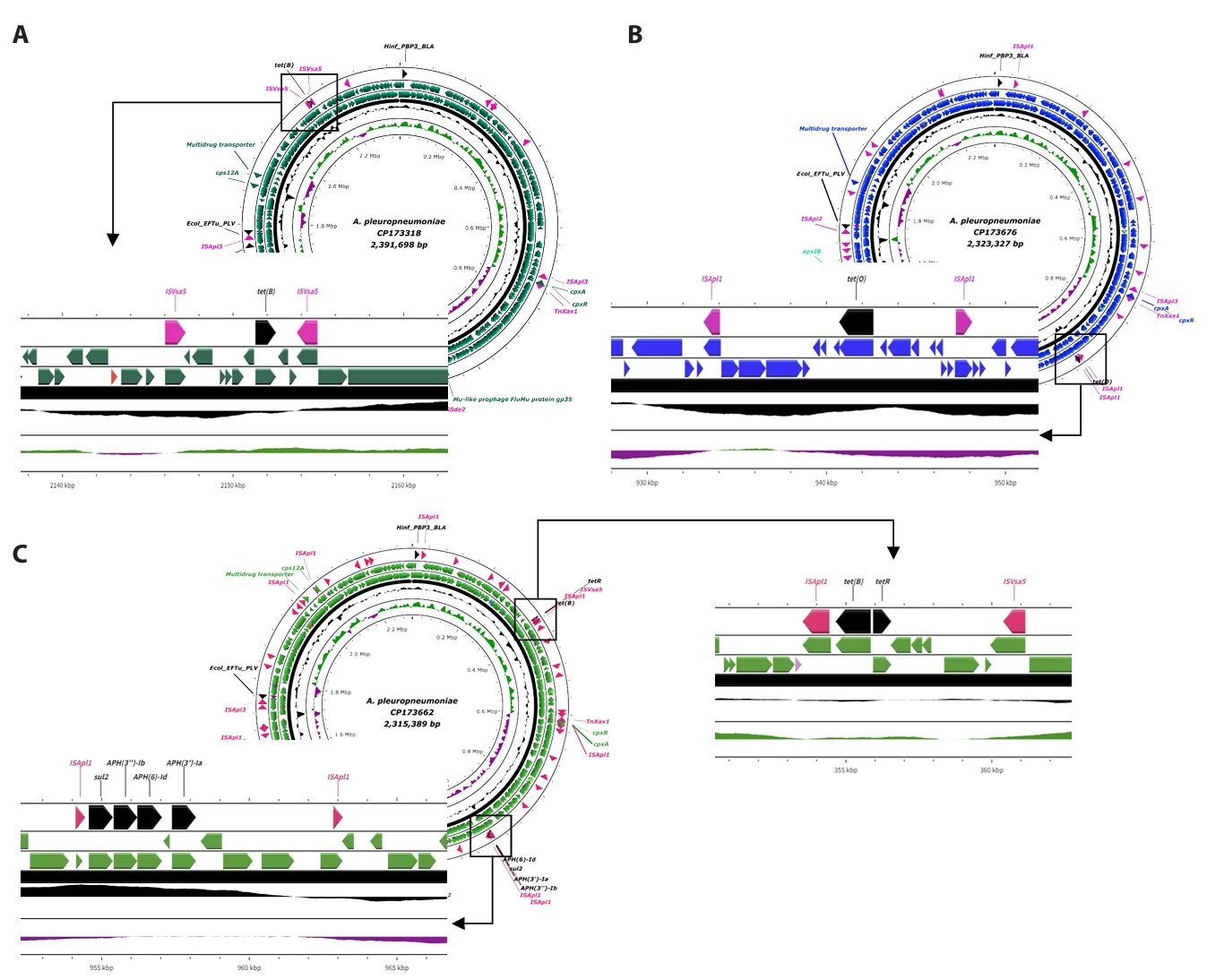
Linear structure of AMR and transposase genes in APSW224, APSW232, APSW234 strains. (A) shows the ISVsa5-tet(B)-ISVsa5 part in CP173318. (B) illustrates the ISApl1-tet(O)-ISApl1 part in CP173676. (C) presents the two parts of AMR genes with mobile genetic elements. The antimicrobial resistant genes are presented as black labels and the mobile genetic elements are indicated with pink labels.

**Fig. 3.**
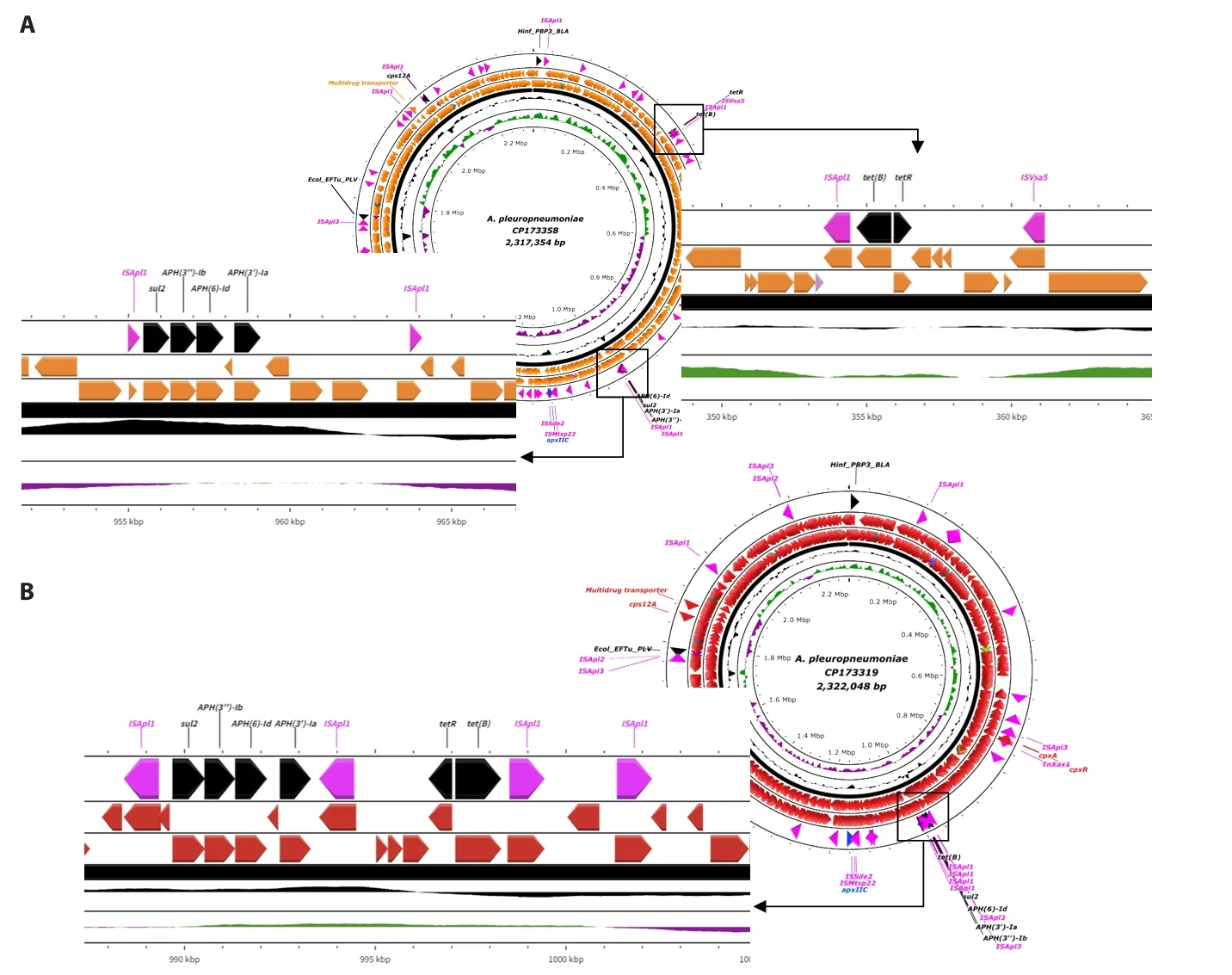
Linear structure of AMR and transposase genes in APSW235, APSW241 strains. (A) indicates the two parts of AMR genes with mobile genetic elements in CP173358. (B) provides the gene cassettes AMR genes and mobile genetic elements in CP173319. The antimicrobial resistant genes are presented as black labels and the mobile genetic elements are indicated with pink labels.

Additionally, the APSW224 strain harbored *tet(B)* gene and APSW232 strain harbored *tet(O)* gene. APSW234, APSW235, and APSW241 strains were found with *tet(B)*, *tetR*, *sul2*, *APH(3'')-Ib*, *APH(6)-Id*, and *APH(3')-Ia* genes. Similar to toxin genes, some transposase genes were detected to be adjacent to some antibiotic resistance genes (Figs. 2 and 3).

Based on the genotyping of antibiotics resistance genes, MIC tests were performed according to the types of resistance genes identified. According to CLSI breakpoint criteria, all isolates except collected in 2024 were resistant to tetracycline. Also, a high level of resistance to sulfamethoxazole was also observed (Table 4).

### Phylogenetic analysis epidemiological profiles

**Fig. 4.**
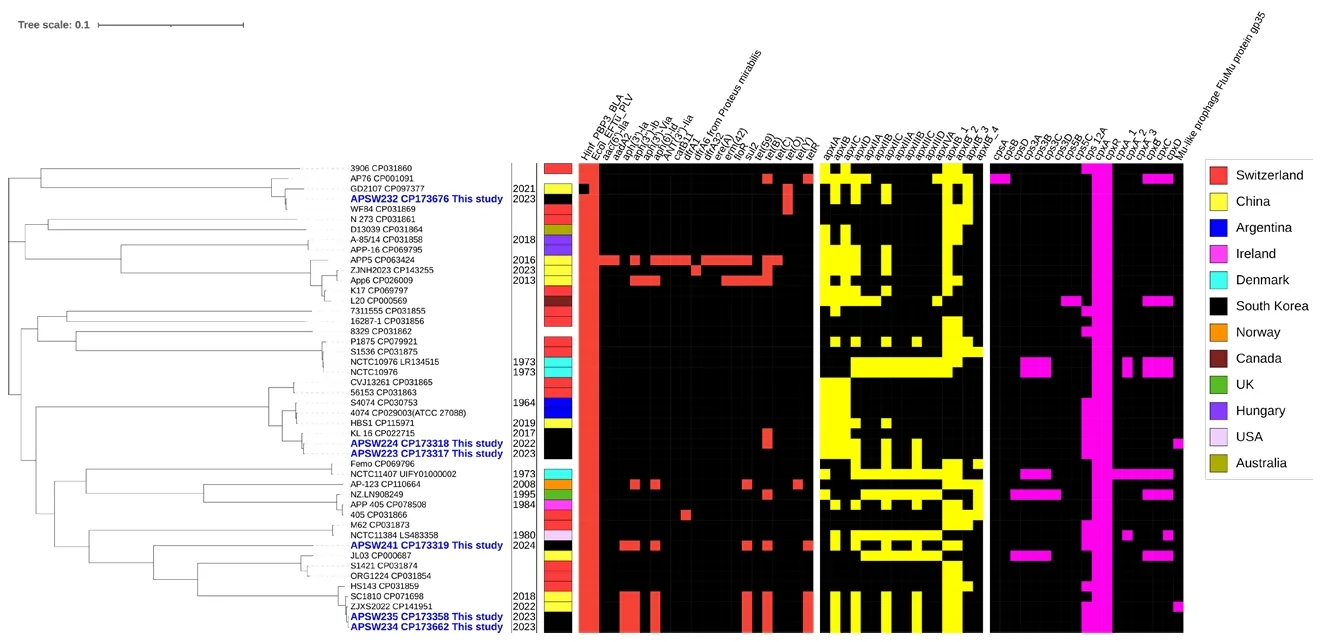
Phylogenetic analysis with 40 global APP isolates. The phylogenetic tree was constructed using 40 global APP isolates to analyze the evolutionary relationships and the distribution of functional genes. The tree was visualized and annotated using the iTOLs (Interactive Tree Of Life) program. The APP strains which were isolated in this study were presented with blue color text. The location information was presented with color strips. AMR genes were displayed with red heatmap. The Apx toxin genes were presented with yellow heat map. The virulence factor genes were indicated with pink color heatmap. Abbreviations: APP, *Actinobacillus pleuropneumoniae*; iTOL, Interactive Tree Of Life; AMR, antimicrobial resistance; Apx, *Actinobacillus pleuropneumonia* toxins.

Epidemiological profiles of total 46 APP strains were presented by phylogenetic analysis (Fig. 4). Isolated year, location and serotype are presented with colored ranges and color strips. Twelve different countries were distributed in the phylogenetic tree and Korean isolates of APP in this study were closely located to China and Switzerland strains (Fig. 4, Table S3). Profiles of *apx* toxins, virulence factors and antibiotic-resistant genes from total 46 APP stains were displayed with heatmap (Fig. 4, Table S4). In this analysis, 23 types of genes were identified. *Hinf_PBP3_BLA* and *Ecol_EFTu_PLV* genes were harbored in almost all strains, followed by a high prevalence of the *tet(B)* gene (Fig 4, Table S4).

## Discussion

APP is a Gram-negative bacteria causing porcine PP, a highly contagious respiratory disease in pigs with significant economic impact. APP is classified into 19 serovars and two biovars, and geographical and temporal differences were described in the its distribution of serotypes and biovars (To et al., 2024). Although several kinds of vaccines and antibiotics have been used to prevent PP, the emergence of new serotype and the increase in antibiotic resistance have necessitated the search for new preventive methods. To address these emerging issues, it is essential to characterize the genetic and epidemiological characteristics of APP field isolates using state-of-the-art analytical techniques. Therefore, six strains were isolated from field emergence cases of PP and whole genome of the isolates were sequenced and analyzed to characterize genomic features and compare with global strains, thereby providing epidemiological insights into swine PP in Korea.

In this study, the results of serotype analysis of domestic field isolates based on the in silico PCR were 1, 7, and 15, which are different from the serotypes previously reported in Korea (Kim et al., 2016), suggesting the introduction of new serotypes into Korea or the emergence of new serotypes due to mutation. However, all field isolates in this study harbored *cps12A*, which was consistent with the results of Switzerland, Argentina, and China on a global scale (Table S4).

Apx toxins, a key virulence factor of APP, exhibit different patterns in the production depending on serotype, and also exhibits differences in virulence due to the difference in hemolysis and cytotoxicity of the toxin. In this study, the possession of the *apx* gene varied depending on the year of isolation. Thus, the emergence of new serotypes and genetic variation in virulence factors of APP field isolates suggest that new challenges must be faced with the currently used control measures. For this purpose, continuous monitoring on the field cases of PP through continuous isolation of APP field strains and genetic and immunological characterization, is essential. Although this study investigated antibiotic resistance in a limited number of field isolates, it demonstrated the potential emergence and spread of novel antibiotic-resistant strains that may challenge eradication of PP in the field.

The genotypic and phenotypic analysis of APP Korean isolates on antibiotic resistance in this study showed that those strains harbored *Hinf_PBP3_BLA* and *Ecol_EFTu_PLV* genes, which are common genes shared by most worldwide isolates of APP. *Hinf_PBP3_BLA* encodes a penicillin-binding protein with beta-lactam resistance enzyme, while *Ecol_EFTu_PLV* gene was originated from *E. coli* with resistance to pulvomycin (Zhang et al., 2024).

Analysis of whole-genome sequences of APP isolates from GenBank revealed that the prevalence of AMR genes, excluding *Hinf_PBP3_BLA* and *Ecol_EFTu_PLV*, has increased globally since the 2010s. A similar phenomenon was observed among the domestic isolates in this study. Specifically, while the APSW223 strain lacked most AMR genes, isolates collected in subsequent years exhibited an increasing number of AMR genes over time. Especially, the APSW234, APSW235, and APSW241 strains harbored large number of antibiotic resistance genes. These strains harbored 6 resistance genes, including *tet(B)*, *tetR*, *sul2*, *aph(3'')-Ib*, *aph(6)-Id*, and *aph(3')-Ia*. Among these genes, *tet(B)*, which exhibits resistance with efflux pump (Santamaría et al., 2011), was detected in recent Korean isolates has also been identified in 8 APP isolates worldwide. Most strains with *tet(B)* were reported in China and Korean with mobile genetic elements, including IS4 family transposase *ISVsa5* and IS30 family transposase *ISApl1*. The gene *tetR*, which encodes repressor of the *tet(A)* gene, and its regulatory interaction controls the expression of *tet(A)* depending on the presence or absence of tetracycline, thereby exhibiting resistance to tetracycline (Ramos et al., 2005). Therefore, *tetR* and *tet(A)* genes are usually found together, but only the *tetR* gene was identified in the field isolates, APSW234 and APSW235, in this study. This is presumed to be due to the transfer of this gene as there are two transposase gene, the IS30 family transposase gene *ISApl1* and the IS4 family transposase gene *ISVsa5*, around *tetR* or the deletion of the previously existing *tet(A)* gene (Figs. 2 and 3). Considering the both tetracycline resistance-related genes and tetracycline MIC results, APSW223 was susceptible, APSW241 showed intermediate susceptibility, and the remaining isolates were resistant. Therefore, the presence of AMR genes was generally correlated with the phenotypic resistance patterns.

The *sul2* - *aph(3'')-Ib* - *aph(6)-Id* - *aph(3')-Ia* genes were commonly identified in strains, APSW234, APSW235, and APSW241, through combinations. Those genes express the resistance to sulfonamide, streptomycin, kanamycin, and neomycin (Ashenafi et al., 2014; Chiou and Jones, 1995; Ramirez and Tolmasky, 2010; Sha et al., 2023), and the resistance was confirmed through determination of MIC against those antibiotics with those APP isolates. Also, the IS30 family transposase gene *ISApl1* is on both sides of these 4 genetic cassettes (Figs. 2 and 3).

Generally, transposase genes associated with the expression of transposons (Babakhani and Oloomi, 2018) were located with the AMR genes. The location of this transposase in the genome, which is located near to the antibiotic resistance gene, was also confirmed in the APP strain isolated in this study. These characteristics were also confirmed in other strains through BLAST analysis. *tet(B)* region in APSW224 identified in *P. multocida* HN07 strain and *S. enterica* subsp. *enterica* serovar Typhi plasmid. The *tet(O)* gene in APSW232 strain was found in APP GD2107 and WF83 strains. The *tet(B)*, *tet(R)*, and transposase regions of strain APSW234 and APSW235 were also identified in *S. enterica* 1559 strain, *G. parasuis* strain EHP1804 and *E. coli* strain AR_0089. The rest of the four AMR gene cassettes, were harbored in *G. parasuis* d76 and GHP1807 strains. In the APSW241 isolates, the combination of *tet(B)*, *tetR* genes and four AMR genes cassettes were found with transposase present position. This similar genetic arrangement was also found in *G. parasuis* YHP 1815, aHPS7 strains, and APP AH2022 strain. BLAST analysis of the antibiotic resistance genes showed that those antibiotic resistance and transposase genes were found in several other strains, suggesting that antibiotic resistance genes are spreading through interspecies horizontal transfer.

According to the phylogenetic analysis in this study, Korean isolates were closely related to strains which were reported previously in China and Switzerland. This genetic association can be predicted to be caused by international movement of domesticated pigs, as well as the distribution of feed, livestock-derived products, or other animal-derived materials that could facilitate bacterial dissemination.

Conclusively, although the number of strains used in this study was small, it was the first report in Korea to analyze the whole genome of APP strains isolated in the past three years, and based on this, it was possible to elucidate the genetic characteristics of Korean field isolates, especially the recent genetic variations in virulence factors and antibiotic resistance. Results from this study could be evaluated as basic knowledge for the prevention and treatment of APP infections, such as vaccine development and antibiotic selection. Furthermore, it is thought that changes in control measures for PP are necessary to appropriately respond to changes in strains through periodic research.

## Figures and Tables

**Table 1.** General properties of isolated APP

Strain	APSW223	APSW224	APSW232	APSW234	APSW235	APSW241
Isolated year	2022	2022	2023	2023	2023	2024
Serotypes	1	1	7	15	15	Unknown
Size (bp)	2,360,280	2,391,698	2,323,327	2,315,389	2,317,354	2,322,048
G + C (%)	41.24	41.23	41.22	41.22	41.22	41.26
CDS (n)	2,222	2,242	2,161	2,129	2,132	2,164
rRNA (n)	19	19	20	19	19	20
tRNA (n)	62	62	61	61	61	61
Resistance related genes (n)	3	4	4	9	9	9
GenBank number	CP173317	CP173318	CP173676	CP173662	CP173358	CP173319

**Table 2.** The status of virulence factors related genes in isolates

Strain	*apxIA*	*apxIB*	*apxIC*	*apxID*	*apxIIC*	*apxIIIC*	*cps 12A*	*cpx A*	*cpx R*	Mu-like prophage *gp35*
APSW223	O	O	O	O	O	-	O	O	O	O
APSW224	O	O	O	O	O	-	O	O	O	O
APSW232	-	O	-	O	O	-	-	O	O	-
APSW234	-	O	-	O	O	O	O	O	O	-
APSW235	-	O	-	O	O	O	O	O	O	-
APSW241	-	O	-	O	O	O	O	O	O	-

**Table 3.** The antibiotics resistance genes based on the Comprehensive Antibiotic Resistance Database (CARD)

Strain	Antibiotics resistance genes								
	*tet(B)*	*tet(O)*	*tetR*	*sul2*	*APH(3'')-Ib*	*APH(6)-Id*	*APH(3')-Ia*	*Hinf_PBP3_BLA*	*Ecol_EFTu_PLV*
APSW223	-	-	-	-	-	-	-	O	O
APSW224	O	-	-	-	-	-	-	O	O
APSW232	-	O	-	-	-	-	-	O	O
APSW234	O	-	O	O	O	O	O	O	O
APSW235	O	-	O	O	O	O	O	O	O
APSW241	O	-	O	O	O	O	O	O	O

**Table 4.** The result of Minimum Inhibitory Concentration (MIC)

Strain	Antibiotics						
	Tetracycline	Doxycycline	Minocycline	Sulfamethoxazole	Streptomycin	Kanamycin	Neomycin
APSW223	< 0.25	< 0.25	< 0.25	32	2	32	32
APSW224	4	0.5	0.5	64	4	32	64
APSW232	32	16	16	4	4	4	1
APSW234	32	32	16	> 256	> 256	> 256	128
APSW235	16	2	0.25	> 256	> 256	> 256	128
APSW241	1	0.25	0.25	> 256	64	16	2

## Supplementary Information

Table S1. The whole genome profiles of sequenced *Actinobacillus pleuropneumoniae* isolates

Table S2. The BUSCOs results of sequenced *Actinobacillus pleuropneumoniae* isolates

Table S3. Profiles of analyzed whole genome datasets in this study

Table S4. Profiles of the antibiotics resistant genes from whole genome of 46 *Actinobacillus pleuropneumoniae* datasets in this study

Table S5. Growth rates and morphological observations of APP on different media types

Fig. S1. Growth curves of six *Actinobacillus pleuropneumoniae* isolates and two reference serotypes (serotype 2 and 5). All strains were cultured in Brains Heart Infusion (BHI) broth supplemented with 15 µg/ml NAD and incubated at 37℃ with shaking. Bacterial growth was monitored by measuring the optical density at 600 nm (OD_600_). The graph illustrates that all tested strains exhibit a typical bacterial growth pattern. Abbreviations: APP, *Actinobacillus pleuropneumoniae*: BHI, Brain Heart Infusion; NAD, nicotinamide adenine dinucleotide; OD_600_, optical density at 600 nm.
