# Supplementary material for: Revealing genetic variation of Actinobacillus pleuropneumoniae Korean isolates using whole genome sequence analysis
Source: J Microbiol. 2026 Apr 21;64(5):e2512010. doi: 10.71150/jm.2512010 (PMC13577021; doi:10.71150/jm.2512010)
Supplement: Supplementary file 1 [file jm-2512010-Supplementary-Table-S5.pdf]

Table S5. Growth rates and morphological observations of APP on different media types

		APSW223	APSW224	APSW232	APSW234	APSW235	APSW241	Serotype 2	Serotype 5
Enriched media	Chocolate agar (V factor O)	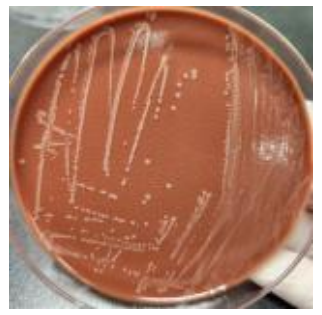	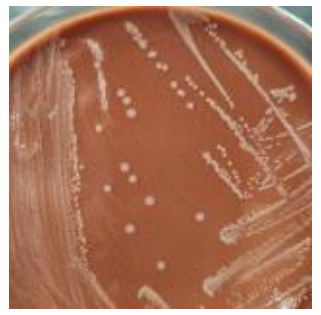	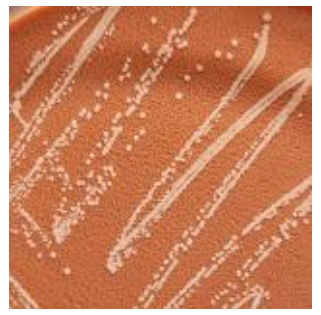	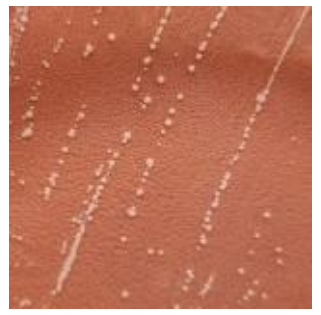	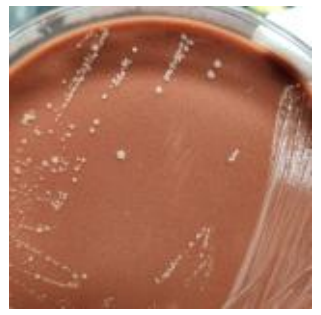	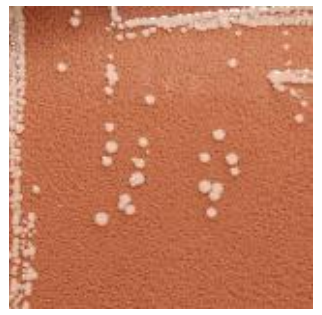	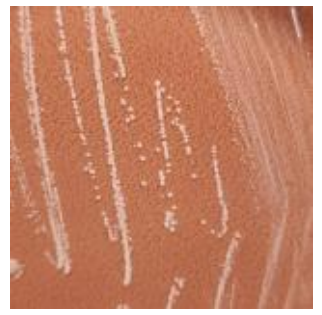	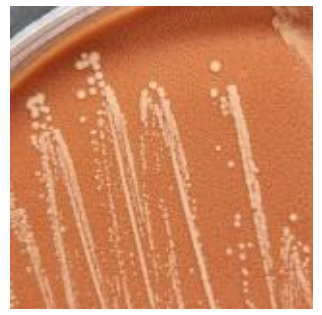
	Blood agar (NAD X)	-	-	-	-	-	-	-	-
Brain Heart Infusion agar	NAD O	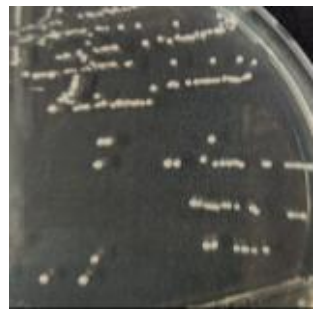	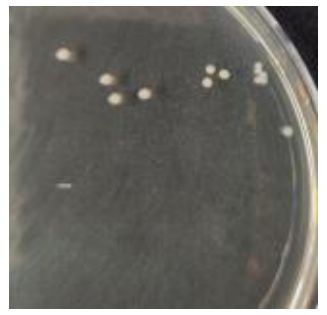	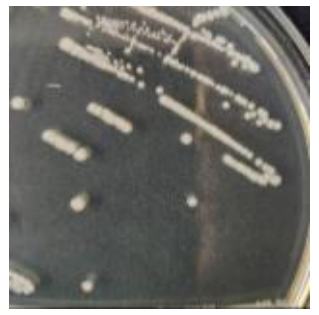	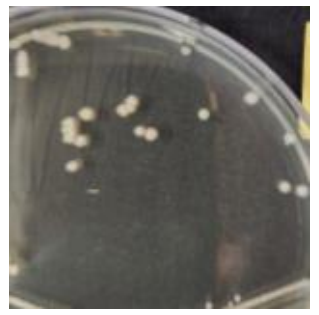	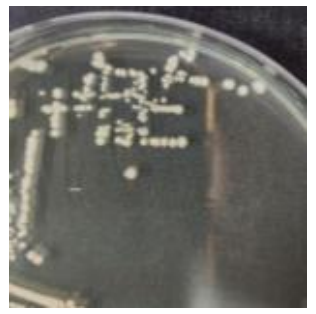	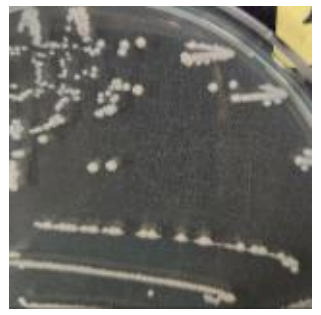	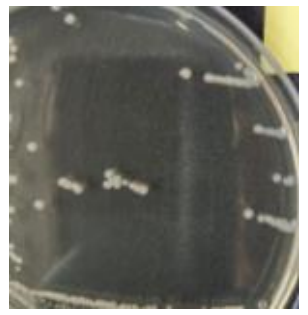	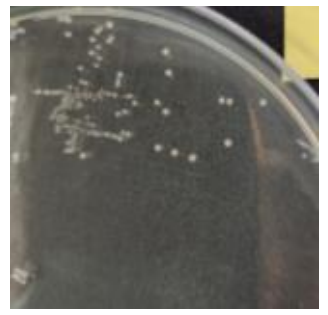
	NAD X	-	-	-	-	-	-	-	-
EMB agar	NAD O	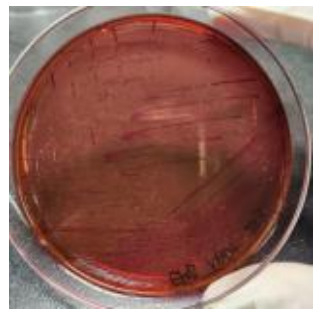	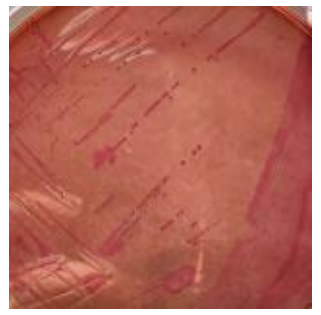	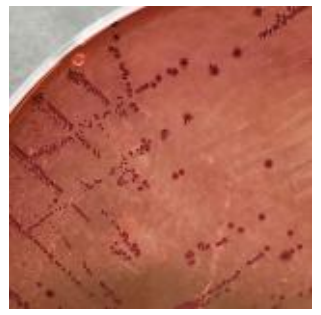	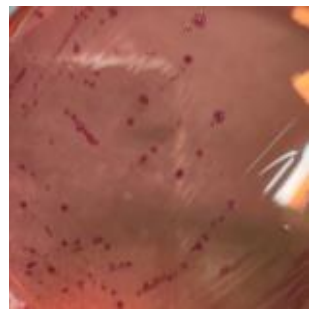	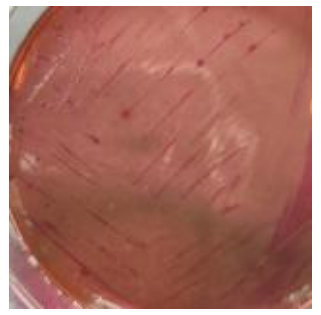	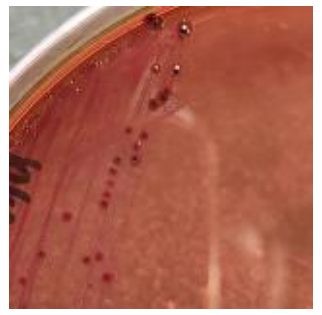	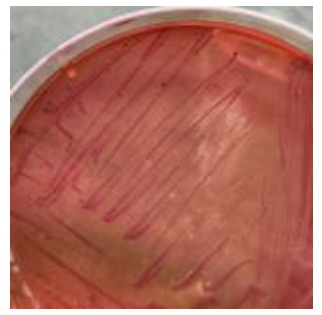	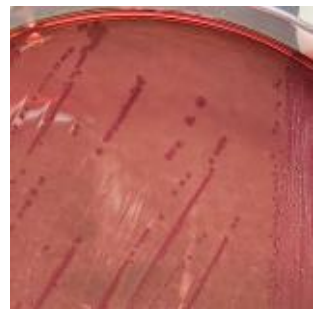
	NAD X	-	-	-	-	-	-	-	-
MacConkey agar	NAD O	-	-	-	-	-	-	-	-
	NAD X	-	-	-	-	-	-	-	-

* - No growth.
